# A weak-labelling and deep learning approach for in-focus object segmentation in 3D widefield microscopy

**DOI:** 10.1038/s41598-023-38490-2

**Published:** 2023-07-28

**Authors:** Rui Li, Mikhail Kudryashev, Artur Yakimovich

**Affiliations:** 1 Center for Advanced Systems Understanding (CASUS), Helmholtz-Zentrum Dresden-Rossendorf e. V. (HZDR), Görlitz, Germany; 2 Max Delbrück Center for Molecular Medicine in the Helmholtz Association, Berlin, Germany; 3 Institute of Medical Physics and Biophysics, Charite-Universitätsmedizin, Berlin, Germany; 4 Bladder Infection and Immunity Group (BIIG), Division of Medicine, Department of Renal Medicine, University College London, Royal Free Hospital Campus, London, UK; 5 Artificial Intelligence for Life Sciences CIC, Dorset, UK

**Keywords:** Computational biology and bioinformatics, Data processing, Image processing, Machine learning

## Abstract

Three-dimensional information is crucial to our understanding of biological phenomena. The vast majority of biological microscopy specimens are inherently three-dimensional. However, conventional light microscopy is largely geared towards 2D images, while 3D microscopy and image reconstruction remain feasible only with specialised equipment and techniques. Inspired by the working principles of one such technique—confocal microscopy, we propose a novel approach to 3D widefield microscopy reconstruction through semantic segmentation of in-focus and out-of-focus pixels. For this, we explore a number of rule-based algorithms commonly used for software-based autofocusing and apply them to a dataset of widefield focal stacks. We propose a computation scheme allowing the calculation of lateral focus score maps of the slices of each stack using these algorithms. Furthermore, we identify algorithms preferable for obtaining such maps. Finally, to ensure the practicality of our approach, we propose a surrogate model based on a deep neural network, capable of segmenting in-focus pixels from the out-of-focus background in a fast and reliable fashion. The deep-neural-network-based approach allows a major speedup for data processing making it usable for online data processing.

## Introduction

Gaining insights into biological processes in three dimensions (3D) is vital for understanding biological mechanisms, as well as improving translation between in vitro and in vivo^1^. However, following the historical concept of microscopy, the vast majority of common techniques used in laboratories remain focused on acquiring 2D images. Among other existing techniques, confocal laser scanning microscopy (CLSM)^2^ remains the most widely used to capture 3D information about biological entities. During CLSM imaging, the pinhole present in the optical path filters out the scattered light, ensuring all the captured intensities are in-focus. This process is repeated for each focal plane as the acquisition moves along the axial axis^3^. In this way, CLSM reconstructs the clear 3D models of biological entities slice-by-slice. Other prominent 3D imaging techniques include the optical section of the specimen in selective plane illumination microscopy (SPIM)^1,4^ and holotomographic microscopy^5–7^. SPIM or lightsheet microscopy typically performs optical sectioning by illuminating the specimen with a sheet of light positioned orthogonally to the imaging path. In fluorescence microscopy, this allows to excite fluorophores only in the focal plane, minimising scatter. Holotomographic microscopy, in turn, employs the holography principle to obtain the 3D image of the specimen^8^. However, longer imaging time, high requirements for trained personnel or facility, as well as high equipment complexity and costs often make these techniques less accessible than traditional widefield microscopy. At the same time, widefield microscopes and binoculars using transmission light are inexpensive, abundant in laboratories across the world, and require minimal training or specimen labelling.

The stepwise acquisition of a larger translucent specimen in 3D may also be performed using widefield microscopy through sequential alteration of the focal plane. Unlike optical sectioning^3^, when widefield microscopy is directly applied to 3D specimens, all light—both in-focus and scattered—contributes to the formation of an image^9^. This introduces the noise from the other focal planes to the recorded images, reduces the contrast information and decreases the quality of 3D reconstruction. An ability to separate in-focus and out-of-focus parts of each focal slice would not only allow for precise 3D reconstruction of the specimen but also make such imaging modality quantitative. This would be possible through a clear separation of background and foreground pixels.

Separation of in-focus and out-of-focus microscopy images without reference may be achieved algorithmically using image-based (passive) autofocusing^10^. In-focus images of specimens are often rich with context patterns which make neighbouring pixels in the in-focus image less autocorrelative compared to the out-of-focus image. This, in turn, leads to greater contrast, wider ranges of intensity, and sharper contour information in the in-focus images, making it possible to evaluate images' in-focus status. Many passive autofocusing algorithms, for example, Vollath^11,12^, Brenner^13^, and Variance^14^ are designed based on this concept. Another more recently proposed approach^15^ employs a discrete wavelet transform^16^, which decreases with the blurring of the image. Yet, most of them are used to evaluate the in-focus status of the whole images or slices in a focal stack. Furthermore, most of these algorithms are relatively slow to compute. This may be addressed using surrogate machine learning models (ML). For example, models based on deep neural networks (DNNs) are recently showing great promise in a plethora of microscopy applications^17–19^. With the development of DNNs has gained more popularity in various computer vision tasks (image classification^20^, segmentation^21^ and object detection, etc.). Through convolution operations, the DNN models extract features from images on multiple scales. These diverse features enhance the accuracy of vision tasks. Specifically, in Waller et al.^19^. have reviewed the potential of DNNs in 3D microscopy^19^. Chen et al.^22^ proposed a 3D convolutional DNN and validated the algorithm for medical image segmentation. Yet, DNN models are known to perform best when used in a supervised ML setting, which would require manual data annotation.

To lower the burden of manual annotation, here we created a novel DNN model for widefield focal sectioning through in-focus pixel segmentation, trained using algorithmically derived Ground Truth (GT). Then, to further improve relevance we employ fine-tuning on the manually segmented GT. It is worth mentioning, that similar approaches to obtain the GT algorithmically have been proposed previously^23–25^. The widefield microscopy image dataset we employed contains in vivo transmission light focal stack micrographs of the *Danio rerio* (zebrafish) larva’s head^26,27^. To obtain the GT we investigated 9 algorithms commonly used in autofocusing tasks including Brenner^13^, Variance^28^, Tenengrad^10,29^, etc. These algorithms obtained focus-score maps of each slice using a sliding window approach and maxima Z-projection. Next, we compared the sensitivity of the focus measurement algorithms using the output of focus measurement algorithms as a focus score. To ensure that the output of these algorithms represents a good proxy for in-focus pixels we compared these outputs to a manually annotated test subset (manually segmented GT). We concluded that five detectors—Variance, Vollath, Standard Deviation^14,30^, Brenner, and Laplacian—were superior to others in detecting changes of focus planes. It is worth noting, that Standard Deviation and Variance, while correlated scale differently in assessing contrast. After the assessment of the target segmentation qualities, we concluded that the Standard Deviation (std) detector outperformed the others in evaluating the focus status of images. Next, we adopted a DNN model with the U-Net^31^ architecture to obtain a surrogate model speeding up the previous focal score computation process.

Our results suggest that using conventional algorithms as weak labels, DNN may be employed as a surrogate model for the detection of in-focus pixels in stable quality. This solution separates the in-focus pixels from image stacks of widefield microscopy, enables the optical sectioning in a digital manner, and reveals the 3D information of the specimen. This, in turn, can make in vivo 3D imaging widely accessible for laboratories with modest funding.

## Methods

### Dataset source and ethics declaration

The dataset of this work comes from the observation of in vivo zebrafish *(Danio rerio)* larvae heads recorded as focal stacks using a stereomicroscope (Leica M205FA; Leica Microsystems, Nussloch GmbH, Nussloch, Germany). All images were obtained at × 130 magnification with a 1 × objective. The lateral resolution was 0.79 μm per pixel. To obtain a focal stack, twenty Z-planes were captured covering a total axial distance of 171 μm at 8.55-μm intervals and saved as TIFF stacks^26^. In each file, the target is in the middle of the view field. As stated in^26^, the animal experiments were performed according to the Animals (Scientific Procedures) Act of 1986 and approved by the Home Office (project licenses PPL P84A89400 and P4E664E3C).

### An algorithm for segmentation of in-focus pixels

The focus measurement algorithms^14^ evaluate the in-focus status through the pixel value patterns in images. Such algorithms give the highest focus score for in-focus pixel intensities. The focus score decreases when the focal plane changes. While a great number of focus detection algorithms have been proposed in the literature (reviewed in paper^28^, including an autofocusing algorithm selection). In this work, we investigate the 9 most widely used algorithms. The algorithms can be classified into three categories based on their design.

### Derivative-based algorithms

These algorithms assume that in-focus images contain more high-frequency content. Therefore, the pixel intensity changes stronger than in out-focus images. These intensity variations can be recognised by computing the derivatives of pixel values. We selected the five most promising algorithms below.

1. Brenner gradient^13^. This algorithm computes the first-order derivation between the target pixel and its neighbours. Equation 1 is presented below with ${(i(x+1, y) - i(x,y))}^{2}\ge \theta$. Here the $\theta$ is a manually defined threshold.
  1 $${F}_{Brenner}={\sum }_{Height}{\sum }_{Width}{(i(x+1,y)-i(x,y))}^{2}$$
  where $x$ corresponds to the pixel position in the horizontal direction, while $y$ indicates the vertical, and *i* corresponds to pixel value (intensity).
2. Tenengrad^29^. This algorithm derives from the Sobel operator by detecting the contour in both horizontal and vertical directions (${S}_{x}(x, y)$ and ${S}_{y}(x, y)$).
  2 $${F}_{Tenengrad}={\sum }_{Height}{\sum }_{Width}{{S}_{x}(x,y)}^{2}+{{S}_{y}(x,y)}^{2}$$
  where $x$ is the pixel position in the horizontal direction and $y$ is vertical. ${S}_{x}$ stands for the Sobel score in $x$ direction, and ${S}_{y}$ means in $y$ direction.
3. Laplacian^32^. This algorithm convolves the image with Laplacian operators and sums the values.
  3 $${F}_{Laplacian}={\sum }_{Height}{\sum }_{Width}\left|{L}_{x}(x,y)\right|+\left|{L}_{y}(x,y)\right|$$
  x corresponds to the pixel position in the horizontal direction, while y indicates the vertical. ${L}_{x}$ stands for the Laplacian score in x direction, and ${L}_{y}$ means in y direction.
4. Sum Modulus Difference (*SMD*)^33^ algorithm calculates the first-order derivation between pixels and neighbours. Here, $F_{SMD}$ represents the SMD score in both $x$ and $y$ directions.
  4 $$F_{SMD} = SMD_{x} + SMD_{y} ,$$
  where $x$ indicates the pixel position in the horizontal direction, and $y$ indicates the vertical.
  $${SMD}_{x}={\sum }_{x}{\sum }_{y}\left|I(x,y)-I(x,y-1)\right|$$
  $${SMD}_{y}={\sum }_{x}{\sum }_{y}\left|I\left(x,y\right)-I\left(x+1,y\right)\right|$$
5. Vollath^11,12^. The Vollath algorithm computes the derivation between pixel intensity in both horizontal and vertical directions.
  5 $${F}_{Vollath}={\sum }_{i=1}^{M-1}{\sum }_{j=1}^{N}g\left(i, j\right)*g\left(i+1,j\right)-{\sum }_{i=1}^{M-1}{\sum }_{j=1}^{N}g\left(i, j\right)*g\left(i+2,j\right),$$
  where $i$ corresponds to the pixel position in the horizontal direction, while $j$ indicates the vertical. $g(i, j)$ stands for the grey-level intensities in position (*i, j*)

### Statistic-based algorithms

These algorithms distinguish the in-focus status by statistical features of images (variance, standard derivation, correlation, etc.). Compared to the derivative-based algorithms, such algorithms are more stable to noise. The candidates in this work are below.

(F) Standard deviation^14,30^. When the images are in-focus, the contrast of pixel values is high. This can be detected by calculating the standard deviation, as shown below
  6 $${F}_{stddev}={\sum }_{Height}{\sum }_{Width}i(x,y)*i(x+1,y)-H*W*{\mu }^{2},$$
  where $x$ corresponds to the pixel position in the horizontal direction, while $y$ indicates the vertical. $H$ and $W$ stand for the height and width of the image. $\mu$ is the mean of the image, and *i* stands pixel intensity.
(G) Variance^14^. Similarly to the standard deviation, variance can also detect the pixel contrast. However specifically to variance, the power operation amplifies the variation differences from pixel values.
  7 $${F}_{var}= \frac{1}{H*W}{\sum }_{Height}{\sum }_{Width}{(i(x,y)-\mu )}^{2},$$
  where $x$ corresponds to the pixel position in the horizontal direction, while $y$ indicates the vertical. $H$ and $W$ stand for the height and width of the image, $\mu$ is the mean of the image.

### Histogram-based algorithm

These algorithms assess the patterns of intensity distributions. This work inspects one histogram-based algorithm outlined below.

(H) Entropy algorithm^34^. This method assumes the in-focus images contain more information about the target. Thus, it shows higher entropy scores.
  8 $${F}_{entropy}=-{\sum }_{Intensities}{p}_{i}*{log}_{2}({p}_{i}),$$
  where ${p}_{i}$ is the probability of pixels with intensity in pixel position $i$.

### Wavelet-based algorithm

(I) Discrete Wavelet Transform (DWT)^15,16^.
  9 $$F_{DWT} = \frac{{\left| {\left| {h_{w} \left( {img} \right)} \right|} \right| }}{{\left| {\left| {l_{w} \left( {img} \right)} \right|} \right|}},$$
  where the *w* in the equation indicates the wavelet. The ${h}_{w}(img)$ stands for the high-pass bands, while the ${l}_{w}(img)$ presents the low-pass band.

### Sliding window scheme

**Figure 1:**
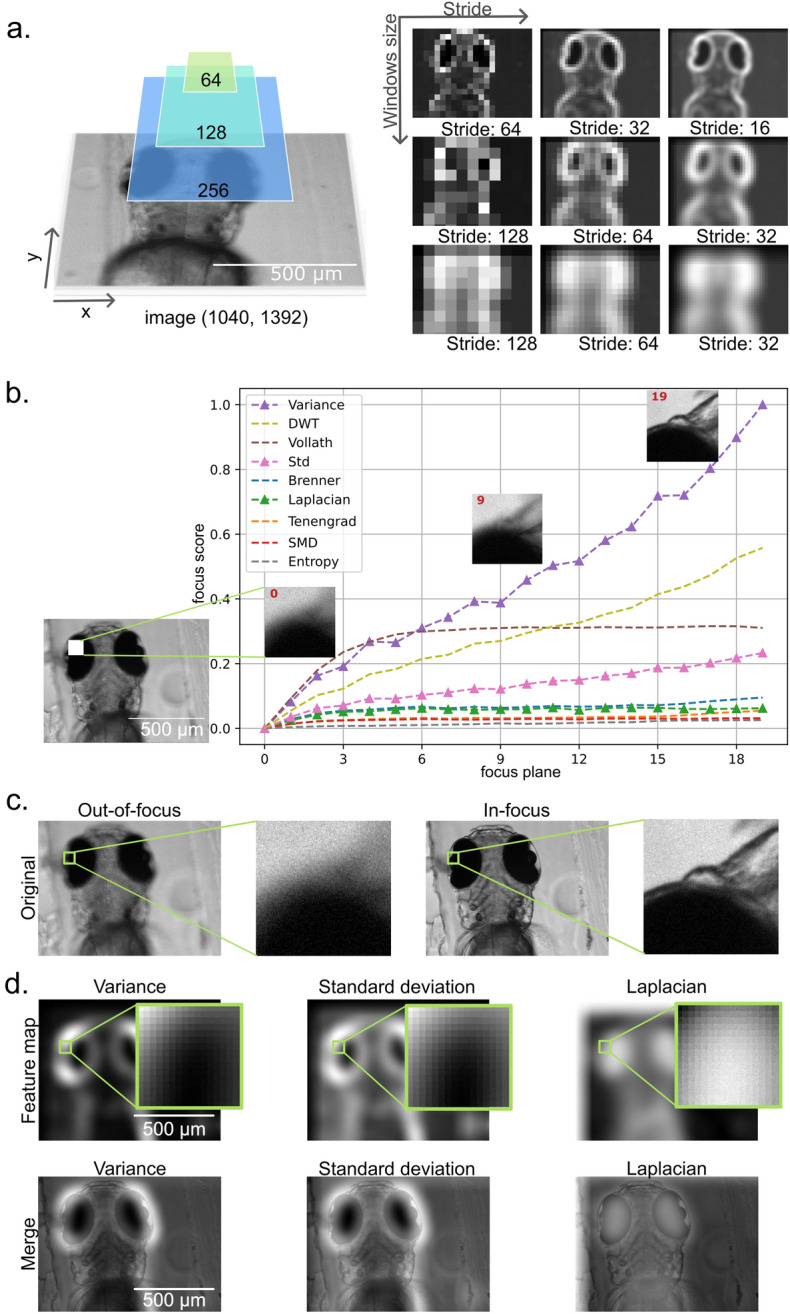
Sliding window scanning approach for focus score maps computation. (**a**) Illustrates a perception window sliding across the widefield microscopy images in both x and y directions, while evaluating the in-focus status of pixels, and outputting the focus-score maps. The three rows show the scanning results with window sizes (64, 128, 256). The focus-score maps with bigger perception windows show fewer details. The three columns stand for different stride plans for specific window sizes. (**b**) Shows the axial sensitivity of focus measurements. The image stack contains 20 images—from the out-of-focus slice 0 to the in-focus slice 19 (shown in the OX axis). Min–max-normalized focus score for each tested algorithm is shown in the OY axis. Figure legend names algorithms in the order of maximum focus score value reached. (**c**) Presents the lateral sensitivity of focus measurements. The slices of one stack range from out-of-focus (left) to in-focus (right). (**d)** The top row shows an example of how the three detectors (Variance, std, and Laplacian) marked the in-focus pixels in the middle slice. This slice contains pixels from both in-focus and out-of-focus. The bottom row shows merges of the maps with microscopy images. The scale bar in all images is 500 µm.

To obtain the focus score maps of the high-resolution micrographs, we employed a sliding (context) window scanning scheme^35^. In this algorithmic scheme, illustrated in Fig. 1a, the context window is moved across the image with a fixed step (stride). For each step the previously described focus scores (9 algorithms described above) were computed on pixels within the context window. In accordance to the respective to the *x* and *y* position of the context window, the values of the respective focus scores were then assembled into the image-wide focus score map.

### Image-wide focus map

The computation of focus scores employing sliding window scheme was repeated on every slice of the image stack in both stride directions (vertical and horizontal). It produces a focus-score map of the scanned image. By changing the stride parameters, this scanning generates multiple focus-score maps with different perceptions. As the sliding windows are calculated on a fixed lattice independently of the vertical or horizontal stride, the results of these calculations are isotropic. Since the in-focus pixels gain higher scores in every scanning, their focus scores peak in the stacks. To obtain the distinct changes for each scanning, we amplify the difference between in-focus and out-focus pixels through the maxima Z-projections of the focus-score stack. This operation preserves the highest focus score for patches. As the scanning repeats, the focus score of in-focus pixels increases more steeply than the out-focus pixels. Finally, we obtain a focus-score map for each slice of the focal stack by reassembling them into a stack and maxima Z-projecting them. This makes it possible to distinguish the in-focus pixels from the images. To diversify the perceptions, the strides parameters and size of windows can be assigned with multiple values as presented in Fig. 1. This compresses the bias introduced by the scanning parameters configurations. Algorithmically, the entire procedure is demonstrated in the following notebook: notebooks/focalMap_demo_update.ipynb, which can be located in our online repository: https://github.com/casus/deepfocus.

### Ranking of the focus score algorithms

Each widefield microscopy dataset contained twenty Z-slices—ranging from in-focus to out-of-focus. Besides the changes of focus status in the lateral plane (Fig. 1a), the focal plane differs also along the axial direction. We proposed the scanning scheme in Fig. 1b. Applied to the different slices, the nine in-focus segmentation algorithms above evaluated the pixels and outputted a focus score for every slice. Since each slice corresponds to different focal planes, the focus scores varied from each other. This makes it possible to distinguish the in-focus slice from the stacks in the axial direction. The higher the focus scores difference between slices, the better the algorithms can recognise the out-of-focus slices.

### Deep neural network architecture

This work proposes a 7-layers symmetric U-Net model with a 3-layer encoder and decoder structure arranged as follows: C256-C128-C64-C32-DC64-DC128-DC256. Here, “C” stands for the convolutional layer, while “DC” stands for the deconvolutional layer. The following number indicates the number of output channels. This U-Net segments the in-focus pixels from the widefield microscopy images end-to-end. This, bypasses calculating the computationally expensive focus score acting as a surrogate model. For the training dataset, we use the previous focus score maps as GT masks. The widefield microscopy image paired with the corresponding GT masks served as input. The U-Net model learns the transfer between raw widefield images and GT masks directly. These GT masks serve as references for in-focus segmentation. After training, the model translates the widefield microscopy image stacks into corresponding 3D pixel information.

### Computations, training and fine-tuning

The GPU calculations for this work were performed on a Tesla V100, and the 9 rule-based algorithms were run on an AMD Rome core. The training speed for our DNN-based solution is 1 s/epoch on average resulting in a training time of 8.5 min for a maximum of 1000 epochs. To avoid overfitting, we implemented early stopping.

Training (pre-training) and fine-tuning were performed using Adam as the optimiser with 0.001 as a starting learning rate. The batch size was 16 and 1 for training and fine-tuning respectively. Early stopping occurred at epochs 425 and 700 for training and fine-tuning respectively. Our datasets consisted of 69 stacks (1380 images) for pre-training on algorithmic GT. Within this dataset we split the stacks with approximately 0.8:0.1:0.1 ratio to obtain training, validation and testing holdouts. For fine-tuning, we used 5 stacks with manually-derived GT (100 images) accompanied by 3 stacks (60 images) for validation and 3 test.

## Results

### Widefield focal stack dataset

To develop an approach for in-focus region detection, we have employed a published dataset of *Danio rerio* (zebrafish) in vivo widefield microscopy^26^. In this dataset, the fraction of the head of the zebrafish is located in the middle of the field of view. Each observation consists of a stack of 20 images taken in different focal planes (focal stack). The last slice (No. 19) of stacks contains mostly in-focus pixels, while most of the pixels in the first slice (No. 0) are out-of-focus. The remaining slices contain a mixture of in-focus and out-of-focus signals (see “Methods” for details). As a result, we have used 69 stacks (1380 images) for pre-training on algorithmic GT, with 0.8:0.1:0.1 split for training, validation and testing holdouts. For fine-tuning, we used 5 stacks with manually-derived GT (100 images) accompanied by 3 stacks (60 images) for validation and 3 test.

### Rule-based in-focus region detection

The widefield microscopy datasets of bulk objects, similar to the one we employed here, often contain in-focus and out-of-focus lateral regions in each slice of the focal stack. These regions change from slice to slice as the focal plane goes through the bulk of the specimen. To distinguish the regions of the slice which are in-focus from those that are out-of-focus, we have explored algorithms typically used for focal plane detection in the axial direction. For each subregion (see sliding window scheme in the “Methods” section) in each slice of the focal stack we have computed a score corresponding to the focal plane detection algorithm (Fig. 1). Specifically, we compared the following 9 algorithms: Brenner, Tenengrad, Laplacian, SMD, Vollath, Std, Variance, Entropy and DWT. To determine the algorithm most suitable to detect lateral in-focus pixels we have used three main criteria: the ability to detect the shift in the axial direction, detail preservation, and computational time.

Figure 1a illustrates the scanning results from one image (slice). To preserve the homogeneity of the original image, this work uses a square perception window (see “Methods” section). As presented in the left part of the panel, the perception window slid in the same step size in both horizontal and vertical directions. We tested the following windows sizes: 64, 128, and 256. We tested the strides (step sizes) 16, 32, 64, and 128 in this experiment. We obtained a focus-score map for each slice of the focal stack by reassembling them into a stack and maxima Z-projecting them (see “Methods”).

As presented in the focus-score map in the right part of Fig. 1a, the brightness indicates a high focus score. We noted that the smaller the perception window was, the more detailed the scanning result was. Yet, a small perception window failed to show the low-frequency information (the global features). For example, the first row extracted only contour information, while the other preserved more global information. Conversely, the large window lost the high-frequency signal (details of images) during scanning leading to undesired results. Notably, between the second and third rows, the third row failed to capture the detailed contour information. Therefore, the balance between low- and high-frequency signals, the window size 128 proved a more appropriate choice for both global features and local details. Next, we examined the stride parameters for this optimal perception window size (Fig. 1a, second row). We noted that the smaller the strides correspond to smoother the final focus scores in the map. A smooth focus-score map indicates the structure details of the zebrafish (eye contour, body components). Conversely, bigger stride steps allow for retaining more global information. This prevents the focus-score map scanning from turning into a simple contour detection method. We further noted that all stride parameters contributed valuable detailed information at various levels. Therefore, a better scanning process should contain multiple stride values to preserve both high-frequency information and local details.

To determine the appropriate focus metric for each region, we applied the nine described rule-based algorithms on the widefield microscopy dataset. The best focus metric was expected to distinguish images on varied focal planes continuously in the axial direction. The in-focus region should score the highest value, while the out-of-focus region should rank at the bottom. With this in mind, we have measured the outputs of each algorithm in comparison to the distance from the perfect focus of a region (defocus). Figure 1b illustrates the sensitivity detection to the focal plane changes.

We noted that six (Variance, DWT, Vollath, Std, Brenner, and Laplacian) out of the nine algorithms detected the focal plane changes successfully—from slice No. 0 to slice No. 19. Interestingly, the Vollath algorithm recognized the difference between in-focus images and out-focus images. However, it failed to detect the changes continuously in the middle slices of the stacks. In these slices, the amount of in-focus pixels was visually comparable to the amount of out-of-focus pixels (mixed-focus slice). However, the Vollath score varied only slightly since the fifth slice (see Fig. 1b). In Table 1, we evaluated the time consumption for all nine candidates. Compared to the other candidates, the Brenner and Vollath algorithms were much more computationally expensive for the same images (16 min/ slice vs. less than 1 min from other candidates). Nonetheless, the results were marginally better than Laplacian. Laplacian, at the same time, preserved a great number of finer structures. Therefore, we concluded that the four algorithms—Variance, DWT, Laplacian, and Std outperform other algorithms in sensitivity along the axial direction.

**Table 1:** The time consumption comparison between 9 candidates for in-focus pixels segmentation.

Candidates	Computation time (s)
Standard deviation (Std)	203
Variance	199
Laplacian	270
DWT	953
Tenengrad	1212
SMD	> 2.4∙10^4^
SMD2	> 2.4∙10^4^
Brenner	1.92∙10^4^
Vollath	1.2∙10^4^
DNN	0.15

To obtain measurements, we evaluated the computation time for segmentation on the whole stack (all 20 slices of one example stack). Noteworthy, SMD and SMD2 correspond to two different implementations of the same algorithm.

Unlike focal plane detection in the axial direction, the task of detecting in-focus parts of the specimen requires focus measurement to distinguish both the in-focus status and the contour information in the lateral directions. The Fig. 1c presents the in-focus status of two images. Zooming into the same patch of these images, the pixel intensities indicate varied contour information. The optimal algorithm should preserve the correct image content during the focus status detection. In the first row of Fig. 1d, we calculated the focus score map from the mixed-focus images (middle slice, mixture from both in-focus and out-of-focus pixels) with the three algorithms above. The detected contour information using the Laplacian differs from the other two. To validate the differences, we merged the focus score map with the corresponding microscopy image in the second row of Fig. 1d. The segmented contour from Laplacian appears to show relatively less detail, compared with Std and Variance.

To avoid dataset bias in this judgement, we have further explored Variance, DWT, Std and Laplacian algorithms performance on synthetic images (Sup. Fig. 1). For this we employed Shepp–Logan phantom (Shepp & Logan 1974) with and without Gaussian blur of variable degree (6 × 6 and 12 × 12 kernel size). The comparison showed that while DWT, Std and Variance algorithms were able to perfectly preserve the low-frequency details, only Std and Laplacian were able to preserve the high-frequency details. Remarkably, Std algorithm was able to preserve both high-frequency and low-frequency details, while computing almost as fast as the Variance—the fastest computing algorithm in our comparison (see Table 1).

**Figure 2:**
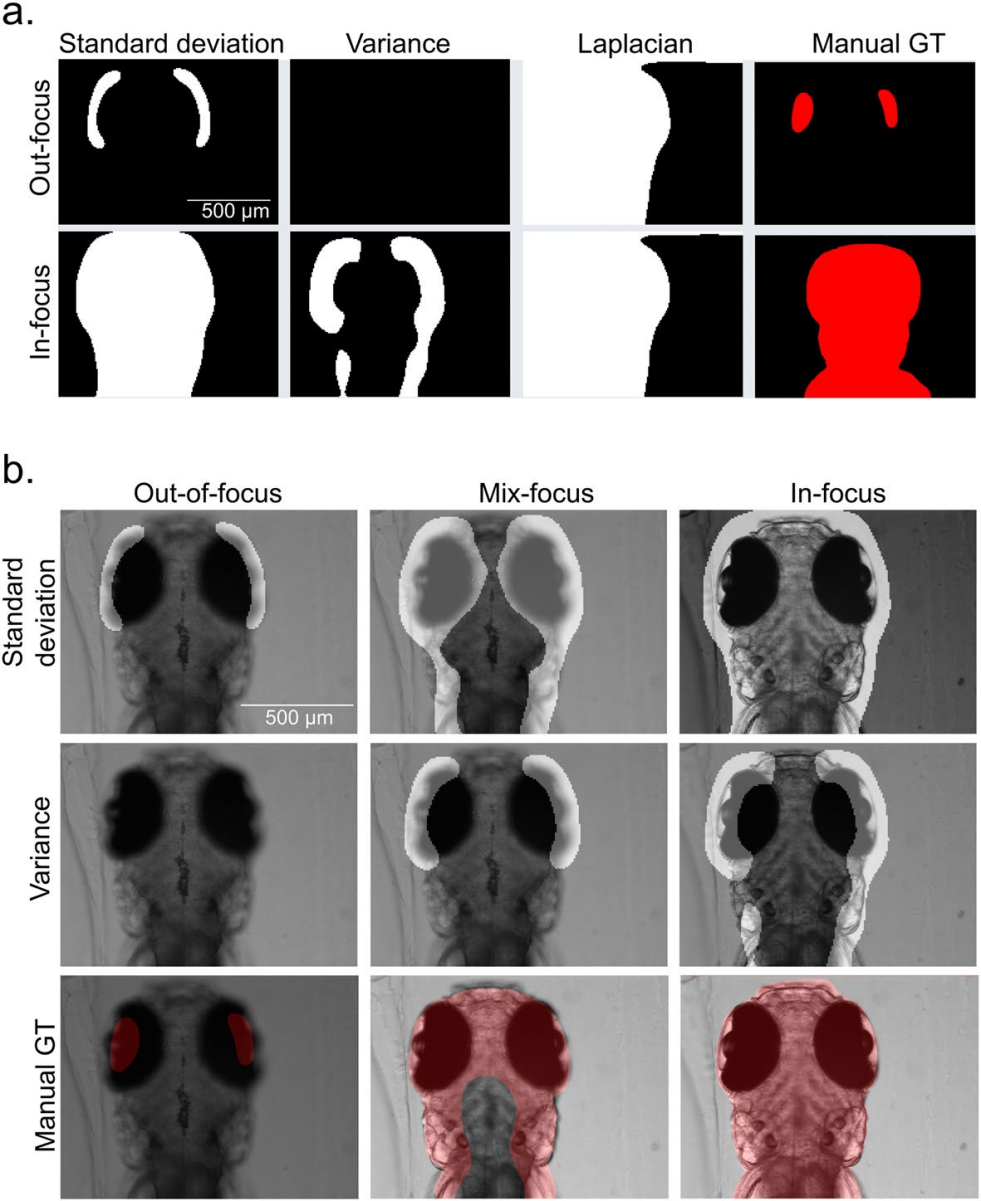
In-focus masks and pixel segmentation. (**a**) Shows binary masks from the focus measurements corresponding to the respective algorithm. To obtain masks, the focus-score maps were binarized using the Otsu thresholding. Comparison with the manually marked ground truth (GT) masks is presented on the right-hand side. (**b**) Shows in-focus masks of Variance and Standard deviation merged with corresponding images compared to the manual ground truth (GT) in red. The scale bar in all images is 500 µm.

Thresholding the focus score map with the Otsu algorithm^36^, we obtained the focus score masks as references for in-focus pixels. This mask preserves the pixels only from the target focal plane and filters out pixels from other focal planes. In Fig. 2a, we compared the segmentations of the three focus algorithms (Laplacian, Variance, and Std) to the manual segmented GT for validation. The Laplacian shows severe inconsistency with the manual GT. Thus, we concluded that it is inferior to the other two in preserving the correct image content. To be noticed, the Variance marks the in-focus pixels in a more conservative way. This yields the loss of specimen information. We assessed the information loss of two algorithms (Variance and Std) on whole stacks in Fig. 2b. Compared to the manual GT, the Variance barely preserved the complete contour information. The Std, however, showed consistency with the manual GT. This makes the Std the focus algorithm of choice satisfying focus sensitivity and the ability to detect the morphology of specimens and fast computation time.

### Deep neural network surrogate for the rule-based in-focus region detection

**Figure 3:**
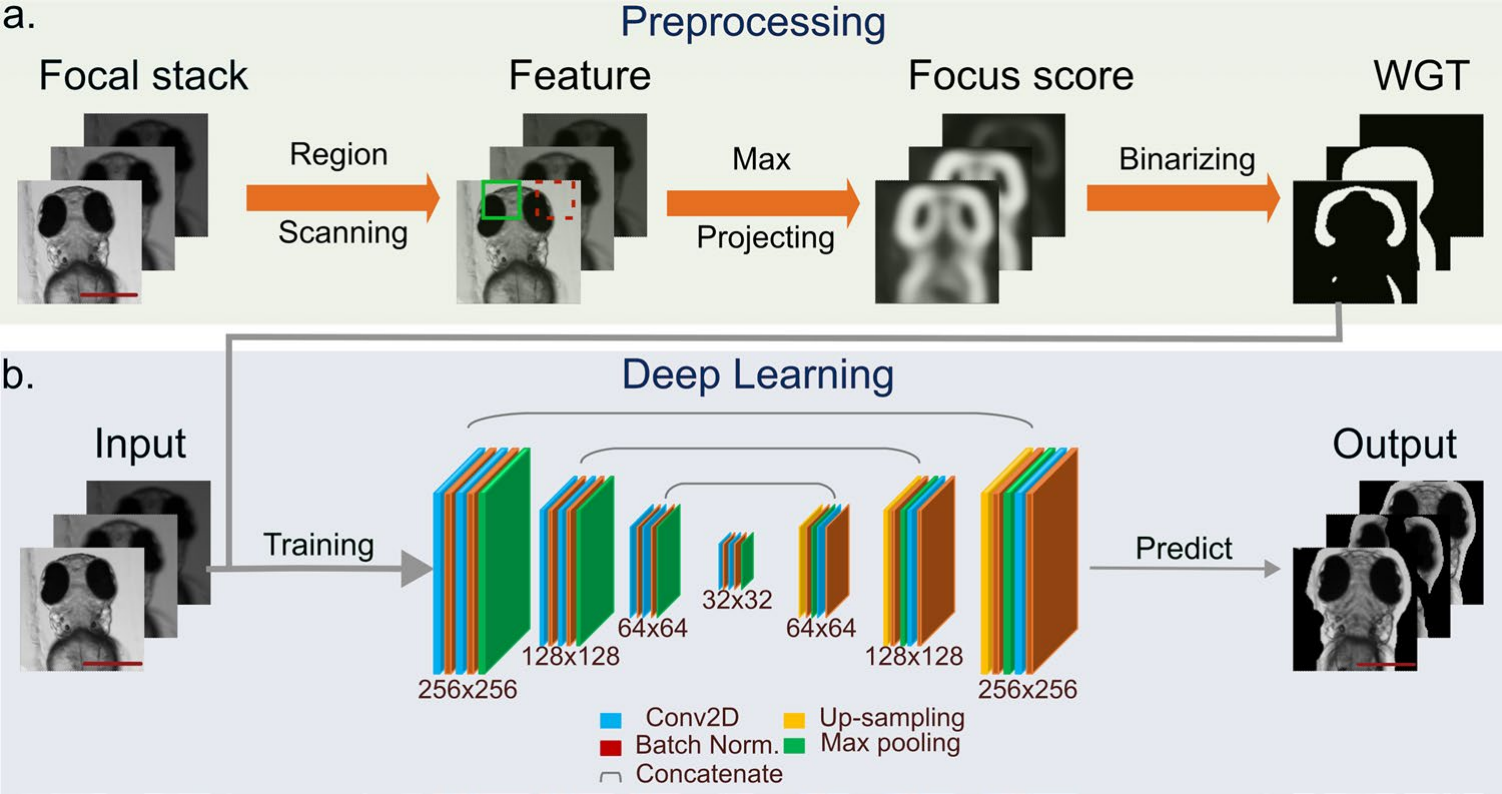
The pipeline of the in-focus segmentation using deep neural networks. (**a**) Shows the pre-processing part, which scans the regions of image stacks from widefield microscopy and outputs the feature map for the maxima projecting. Here, each slice of the z-stack is processed separately. The focus-score map resulting from maxima projecting serves as the input for the binarizing step. This process marks the in-focus pixels in the focus-score maps as the Weak-label Ground Truth (WGT) masks. (**b**) The deep learning part adopts the WGT masks along with the widefield microscopy images for the surrogate deep neural network (DNN) training. The DNN segments directly the in-focus pixels from the original image stacks and presents the 3D information of targets. The scale bar in all images corresponds to 500 μm.

We have shown that the focus score pipeline with the Std algorithm may generate reliable focus score maps (Fig. 3a). Furthermore, accompanied by automated thresholding algorithms, these maps allow obtaining focus masks which are comparable to the manual GT. However, this rule-based pipeline is relatively computationally expensive and requires a long time to process image stacks). To achieve similar results in an end-to-end single-step fashion, we proposed a DNN surrogate model with the U-Net-like structure^31^ Illustrated in Fig. 3b. To obtain the U-Net-based surrogate model, we used the binary masks captured from the output of the rule-based focus score algorithms as weak labels^24^. This way, the model learns directly the transformation between focus score masks and raw images. Further improvements may be obtained by fine-tuning on manually annotated data (see “Methods”).

We opted for U-Net architecture as it is commonly used in biomedical image segmentation tasks. U-Net combines the convolutional neural network (CNN) and the Autoencoder (AE) like structures^37^. As a representation learning model, the first several convolutional layers of U-Net (the encoder part) enhance channel numbers of the input images and extract the features in the AE structure. The middle convolutional layer (the bottleneck part) encodes the previous features as embedding vectors in the latent space. The last multiple de-convolutional layers (the decoder part) upsamples the embedding vectors back into the original images. Optimizing the loss between reconstructed images and inputs, the encoder and decoder learn jointly the manifold structures^38^ of given datasets. In contrast to the traditional AE structures, the U-Net concatenates the up-sampled embedding code with the feature maps from the corresponding layers in the encoder part^39^. This operation casts constraints on the outputs and gives the U-Net an advantage in the supervised learning tasks. This model segmented the in-focus pixels directly from the widefield microscopy images.

As illustrated in Fig. 3b, this model contains 7 convolutional layers—3 for the encoder; 1 for the bottleneck; 3 up-sampling layers for the decoder. The loss function we chose consisted of focal loss, dice loss, and binary cross entropy summed up into a total loss. To evaluate the performance of the model, this work adopted the IoU score^40^ as the metric. As the optimiser, we used Adam with a learning rate of 0.001. After 400 epochs of learning with a batch size of 8, the model converged to a stable value both for IoU scores and the loss. The final IoU for the validation set was around 0.93 (algorithmic GT). Noteworthy, training augmentations including image flip and 90 degrees rotation, chosen to avoid introducing new pixels or disturbing morphology, improved our performance only marginally (Sup. Fig. 2). As presented in Table 1, this DNN model speeds up the segmentation process with 0.15 s for one stack. Even with the training time of 8.5 min in one shot, this solution is still superior to other candidates by accelerating the segmentation to at least ~ 1000 times. Noteworthy, this gain comes at the cost of accuracy in comparison to the algorithmic GT. Yet, given that the manually-derived GT constitutes the true target, the DNN approach allows for performance improvement through fine-tuning. This notion is often utilised by the weak-labelling approach^24^.

### Surrogate model evaluation

**Figure 4:**
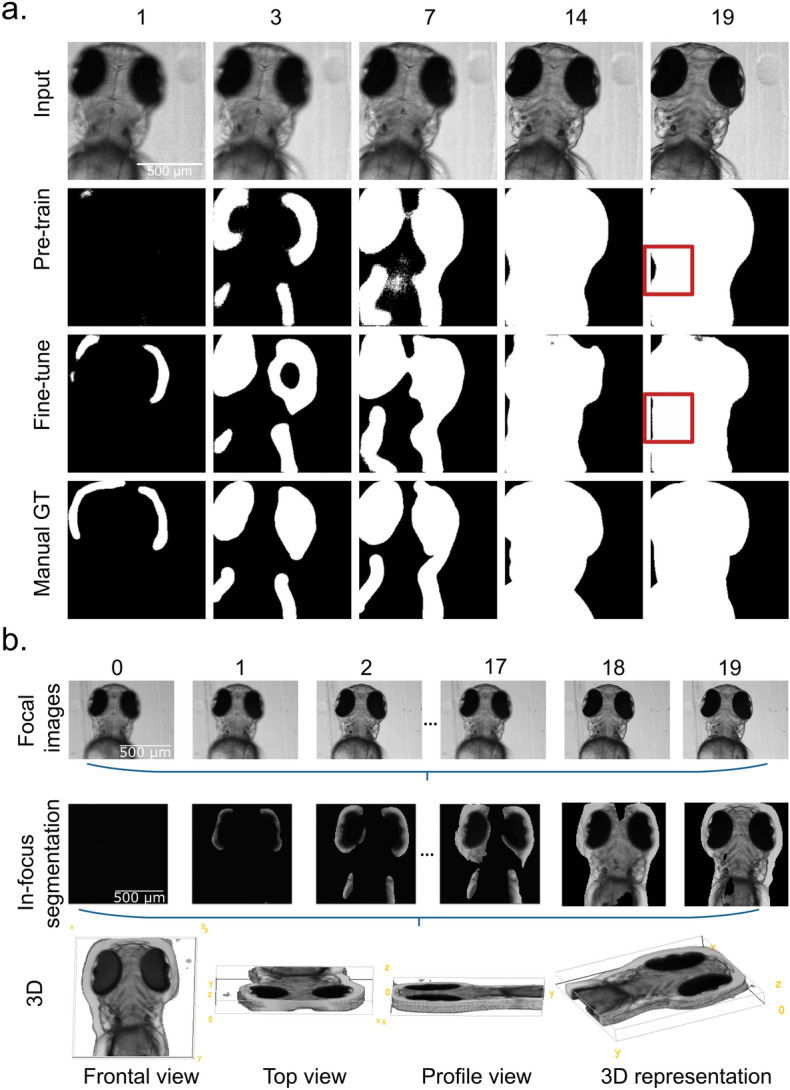
The end-to-end in-focus segmentation model using deep neural network (DNN). (**a**) Shows input, pre-training prediction, fine-tuning prediction, and manual Ground Truth on slices ranging from 1 (out-of-focus) to slice 19 (in-focus) in one stack. Red square denotes region of prediction improvement upon fine-tuning. (**b**) Depiction of the end-to-end pipeline: the trained DNN segments the in-focus pixels from the image stacks of widefield images directly. These pixels represent the 3D information of targets. This enables the operations of optical sectioning in a digital way by using widefield microscopy.

The pre-trained U-Net surrogate model can directly predict the focus-score mask with the widefield microscopy images as inputs. This approach significantly simplified the segmentation compared to the previous focus-score pipeline. Figure 4a illustrates part of the segmentation results. Notably, from the completely out-of-focus slice (slice 0) to the completely in-focus slice (slice 19), the DNN model distinguished the in-focus pixels correctly. In slice 0, the model labelled the whole Image as out-focus. The results were largely consistent with the GT mask. As the focal plane changed during optical sectioning, the image contained more in-focus pixels. The predictions of the DNN model stayed reliable. In slice 19, the model labelled correctly the whole target as in-focus. However, the model trained on algorithmic GT (pre-training) showed minor discrepancies with the manually-derived GT (Fig. 4a, Red Square). This observation was consistent with the performance metric. When validated on the manual GT validation performance of the model trained on the algorithmic GT dropped from 0.832 to around 0.660 using the test holdout.

To further improve the performance of our surrogate model we have fine-tuned it on an unseen portion of 5 manually annotated image stacks containing in total 100 images (Table 2). Remarkably, in such a setting, fine-tuning provided a significant improvement in performance on manual GT from 0.660 to 0.784. At the same time, the performance of the fine-tuned model on the algorithmic GT dropped only insignificantly (Table 2), illustrating that the model training of Std-derived GT can be useful as a pretraining step. Finally, a visual comparison of the fine-tuned model to the manually-derived GT (Fig. 4a, Red Square) suggested higher consistency.

**Table 2:** Model performance pre and post fine-tuning.

Models/test	Algorithmic ground truth	Manual ground truth
Pre-training	0.832	0.660
Fine-tuning	0.802	0.784

The focus-score masks obtained from the surrogate model may be employed for in-focus pixel segmentation from the widefield microscopy images. As shown in Fig. 4b, these masks allow for retaining only the in-focus part of the image. These, in turn, may be assembled into a 3D model of a specimen. Notably, this is possible by employing images obtained using widefield in vivo microscopy, in which unlike in CLSM, both in-focus and out-focus light contribute to the formation of the image in every image plane.

## Conclusion and discussion

Inspired by the concept of 3D object reconstruction from the focal stacks and software-based autofocusing algorithms, this paper proposed an approach to filter in-focus from out-of-focus regions of the image in the focal stack obtained in widefield microscopy. The latter can be obtained by altering the focal plane and scanning through bulk specimens like live zebrafish (*D. Rerio*). However, focal stacks obtained by widefield microscopy in such a manner contain a mixture of information produced by both in-focus and out-of-focus light. To select the optimal focus measurement algorithm, this work investigated nine candidates widely used in software-based focal plane detection (Vollath, Brenner, std, etc.). Our experiments showed that for the purpose of in-focus region detection and content information preservation, standard-deviation-based pipelines were optimal.

To overcome the computational costs of the rule-based pipeline, we proposed a DNN surrogate model based on U-Net architecture for in-focus pixel segmentation. This model was trained to adopt the previous rule-based segmentation results as GT. The resulting DNN model filtered out the out-of-focus signals digitally without a complex and expensive confocal setup. The segmentation results on the zebrafish dataset showed consistency with the manual segmentation GT. Compared to the previous nine candidates, the DNN model outperforms others in the calculation speed being at least ~ 1000 times faster by in-focus segmentations. This may likely be attributed to the highly parallelised nature of the TensorFlow library. We argue that through such an impressive performance virtual optical sectioning employing surrogate DNN may be well-suited for in vivo widefield microscopy. Upon segmentation of the in-focus pixels, our DNN allows us to reconstruct 3D models of the specimen obtained from widefield imaging.

While multiple tasks in machine learning and deep learning for microscopy have been proposed in the past^17,41–43^, no tasks for the separation of in-focus from out-of-focus images have been explored until now. We argue that our work opens an avenue to advanced image protocols, such as 3D in vivo imaging using simple and inexpensive hardware. Widefield microscopes are abundant in research and education facilities and may find new applications using approaches akin to ours. Remarkably, as the GT for our surrogate model was obtained purely programmatically, it is tempting to speculate that this approach may be useful in weak labelling and self-supervised learning^24,44,45^.

As a possible extension of this work, a better focus-score pipeline could combine multiple focus measurement algorithms instead of only one. This could possibly enhance the quality of the segmentation. This serves as a better GT for training the DNN models. Besides, other DNN structures (pix2pix GAN, transfer learning, 3D U-Net, etc.) might bring better performance regarding segmentation accuracy.

## Supplementary Information

Supplementary Figures.

## Data Availability

The program code used in this work is available for use and re-use under an open-source license and can be accessed via GitHub (https://github.com/casus/deepfocus). The Dataset of *D. Rerio* focal stacks was previously published in^26^ and raw or additional data is available upon request from the corresponding authors^26^. As stated in^26^, the animal experiments were performed according to the Animals (Scientific Procedures) Act of 1986 and approved by the Home Office (project licenses PPL P84A89400 and P4E664E3C). Processed data necessary to reproduce this work is available via GitHub repository.
