# Supplementary material for: A weak-labelling and deep learning approach for in-focus object segmentation in 3D widefield microscopy
Source: Sci Rep. 2023 Jul 28;13:12275. doi: 10.1038/s41598-023-38490-2 (PMC10382522; doi:10.1038/s41598-023-38490-2)

Supplementary figures

Supplementary Figure 1. In-focus region detection in Shepp–Logan phantom with varying degrees of blurring. Here synthetic dataset is subjected to varying degrees of Gaussian blurring (6x6 and 12x12), shown in rows. Performance of selected algorithms including discrete wavelet transform (DWT), Standard Deviation (Std), Variance (Var) and Laplacian.

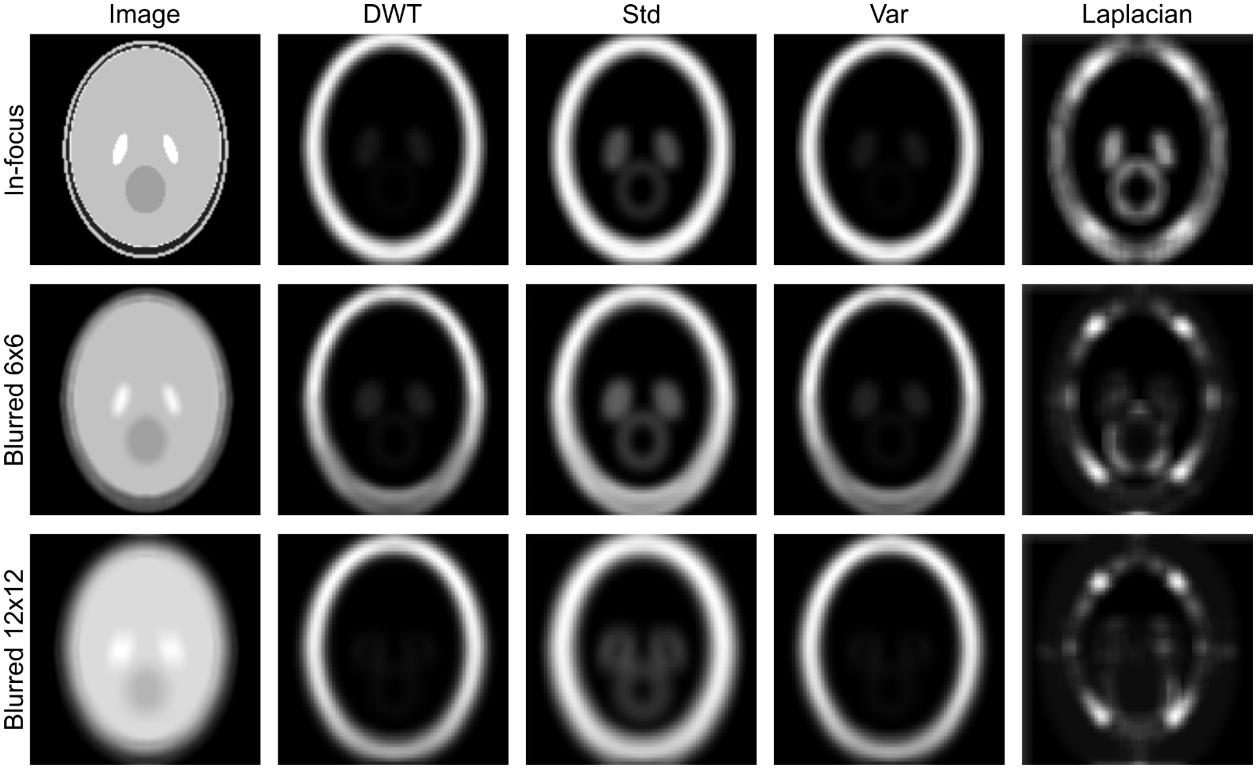

Supplementary Figure 2. Effect of training data augmentation on the model training. The graph shows training performance measured as intersection over union (IoU) with and without image data augmentation. To avoid introducing new pixels 90 degrees rotation and vertical or horizontal flip were used as the augmentation strategy.

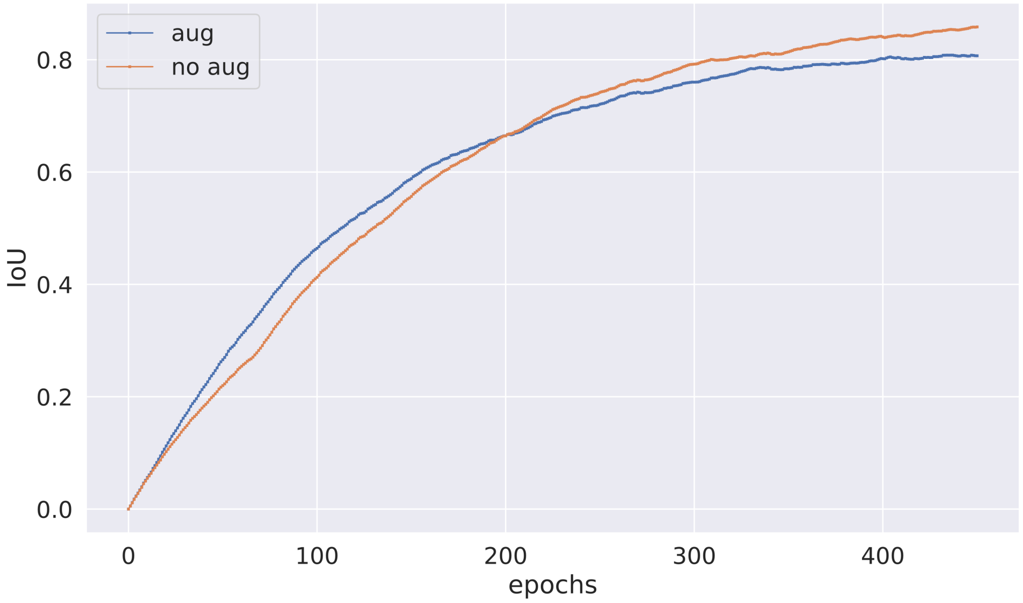

Supplement: Supplementary file 1 — Supplementary Figures. [file 41598_2023_38490_MOESM1_ESM.pdf]
